# Two ω-3 FADs Are Associated with Peach Fruit Volatile Formation

**DOI:** 10.3390/ijms17040464

**Published:** 2016-03-29

**Authors:** Jiao-Jiao Wang, Hong-Ru Liu, Jie Gao, Yu-Ji Huang, Bo Zhang, Kun-Song Chen

**Affiliations:** 1 Zhejiang Provincial Key Laboratory of Horticultural Plant Integrative Biology/Laboratory of Fruit Quality Biology, Zhejiang University, Hangzhou 310058, China; flora_wang@zju.edu.cn (J.-J.W.); hear2008dream@163.com (H.-R.L.); gaoj@163.com (J.G.); akun@zju.edu.cn (K.-S.C.); 2 College of Horticulture, Fujian Agriculture and Forestry University, Fuzhou 350002, China; yjhuang2004@163.com

**Keywords:** fatty acid desaturase, fatty acid, peach fruit, volatiles

## Abstract

Aroma-related volatiles, together with sugars and acids, play an important role in determining fruit flavor quality. Characteristic volatiles of peach fruit are mainly derived from fatty acids such as linoleic acid (18:2) and linolenic acid (18:3). In the present study, six genes encoding fatty acid desaturases (FAD) were cloned, including two ω-6 FAD genes (*PpFAD2*, *PpFAD6*) and four ω-3 FAD genes (*PpFAD3-1*, *PpFAD3-2*, *PpFAD7* and *PpFAD8*). Heterologous expression of peach FADs in tobacco plants showed that *PpFAD3-1*, and *PpFAD3-2* significantly reduced contents of 18:2, and accumulated significant higher levels of 18:3. In the case of volatiles, transgenic plants produced lower concentrations of hexanal and higher levels of (*E*)-2-hexenal. Consequently, the ratio of the (*E*)-2-hexenal and hexanal was about 5- and 3-fold higher than that of wild type (WT) in *PpFAD3-1* and *PpFAD3-2* transformants, respectively. No significant changes in volatile profiles were observed in transgenic plants overexpressing the four other peach FAD genes. Real-time quantitative polymerase chain reaction (qPCR) analysis showed that ripe fruit had high *PpFAD3-1* and low *PpFAD3-2* transcript levels. In contrast, high *PpFAD3-2* and low *PpFAD3-1* transcript levels were observed in young fruit. These results indicate a temporal regulation of these two ω-3 FADs during development and ripening, influencing peach fruit volatile formation.

## 1. Introduction

The aroma of peach (*Prunus persica* L. Batsch) fruit is an important organoleptic property that impacts consumers’ preference. Among more than 100 volatiles that have been identified from peach fruit [1], the compounds derived from fatty acids make a major contribution to sensory quality [2]. These volatiles include C6 aldehydes, alcohols, esters, and lactones. In general, polyunsaturated fatty acids (PUFAs), such as linoleic acid (18:2) and linolenic acid (18:3), are the main precursors for aroma-related volatiles of peach fruit generated via the lipoxygenase (LOX) pathway or β-oxidation [3]. For the LOX pathway, 18:2 and 18:3 are firstly oxidized to the fatty acid hydroperoxides, which are subsequently cleaved by hydroperoxide lyase to form hexanal and hexenal, respectively. Then, C6 aldehydes are reduced to corresponding C6 alcohols by alcohol dehydrogenase and subsequently converted to esters by alcohol acyltransferase. Acyl-coenzyme A (acyl-CoA) oxidase is the first enzyme involved in β-oxidation of fatty acids and was suggested to be associated with lactone formation during peach fruit postharvest ripening [4]. It has been reported that the addition of exogenous PUFAs led to an increase in hexanal and (*E*)-2-hexenal of fruit [5,6,7], implying that modification of fatty acid composition may affect fruit volatile profiles.

In plants, there are two distinct pathways for fatty acid (FA) synthesis: the prokaryotic and eukaryotic pathways. The prokaryotic pathway refers to synthesis of lipids within the plastid, while the eukaryotic pathway refers to synthesis occurring in the endoplasmic reticulum (ER). It has been reported that chloroplast-synthesized fatty acids are exported to the ER, and about half of these exported fatty acids return to the plastid after they enter the eukaryotic pathway [8]. The desaturation of FAs is catalyzed by fatty acid desaturase (FAD), which is associated with fatty acid compositions of plants. As with the FA synthesis pathway, FADs are located in either the chloroplast or the ER. In *Arabidopsis*, conversion from 18:1 to 18:2 is catalyzed by FAD2 and FAD6, which are ω-6 fatty acid desaturases, while ω-3 fatty acid desaturases (FAD3, FAD7, and FAD8) convert 18:2 to 18:3. It has been reported that the desaturation catalyzed by FAD2 and FAD3 takes place in the ER, while FAD6, FAD7, and FAD8 localize to the chloroplast [9].

Silencing or overexpression of FADs showed that modifications of FA composition impacts plant responses to stresses [10,11,12]. Moreover, members of the FAD family that are associated with oil quality have been characterized in soybeans [13,14] and olives [15,16,17]. However, studies of FADs related to plant volatile profiles are relatively limited, mainly to the model fruit tomato. The tomato *lefad7* mutant showed an increase of 18:2 and a decrease of 18:3, accompanied with reduced contents of (*Z*)-3-hexenal and (*Z*)-3-hexenol [18]. Heterologous expression of *FAD3* and *FAD7* in tomato also influenced FA composition and volatile profiles [19]. Beyond the tomato, the role of plant FADs associated with volatile formations has not been extensively studied. In our previous study, four FAD genes were cloned, and their expression profiles were investigated during peach fruit storage [20]. Based on correlation analysis between volatiles and gene expression data, *PpFAD1B_6* was suggested to be involved in the production of a precursor of lactones/esters [21]. Recently, a high-quality draft genome of peach has been released [22], providing an opportunity to further study the physiological roles of the various FADs during fruit development and ripening, particularly in terms of fatty acid compositions and volatile formation.

In the present study, changes in concentrations of volatiles and fatty acids were analyzed during peach fruit development and ripening. Six FAD genes were cloned, followed by expression analysis using real-time quantitative PCR (qPCR). In addition, peach FAD genes were overexpressed in tobacco plants (*Nicotiana tabacum*) to determine modifications in contents of FAs and green sensory volatiles. The physiological roles of peach FAD family are discussed.

## 2. Results and Discussion

### 2.1. Volatile Production during Peach Fruit Development and Ripening

According to the odor descriptors, volatile compounds in peaches can be divided into several sensory groups, including green, fruity and peach-like aromas [23]. Distribution of the volatiles showed that aroma-related volatiles in skin were significantly higher than those observed in the other parts of the fruit [24]. Therefore, peach fruit were sampled at different development and ripening stages followed by analysis of volatile concentrations in skin.

**Figure 1:**
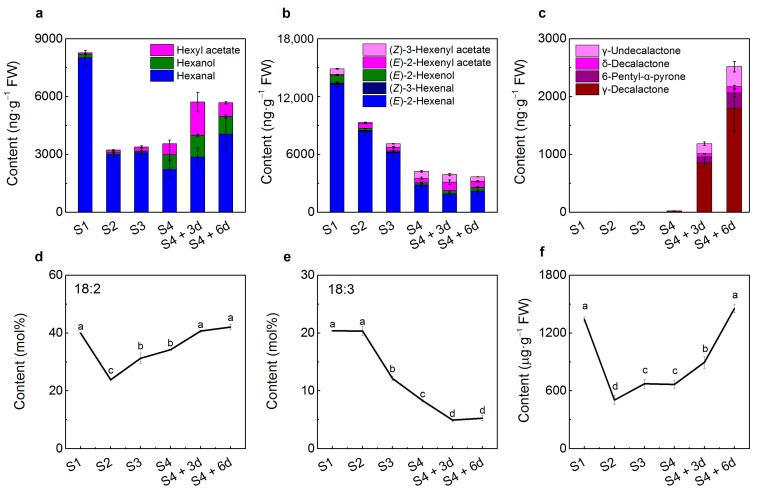
The contents of peach fruit volatiles and fatty acids during development and ripening. (**a**) Contents of volatile compounds derived from 18:2; (**b**) volatile compounds derived from 18:3; (**c**) lactones from fatty acids; (**d**) content of 18:2; (**e**) content of 18:3; (**f**) content of total FAs. Relative quantification of volatile compounds was performed using the peak area of the internal standard as a reference. Data are the mean ± stand error from three replicates. Values indicated by different letters represent significant difference at level of 0.05.

The green sensory volatiles of peach fruit consist of hexanal, (*E*)-2-hexenal, (*Z*)-3-hexenal, and their corresponding alcohols. Progressive declines were observed for both hexanal and (*E*)-2-hexenal content during peach fruit development and ripening, decreasing by 64% and 85% at the third day (3d) of shelf-life after maturity stage (stage 4, S4) as compared to the first fast growth stage (stage 1, S1), respectively (Figure 1a,b). Peach fruit at the S1 stage showed the highest concentrations of (*E*)-2-hexenol, decreasing by nearly 4-fold at S4. In the case of fruity esters, contents of hexyl acetate and (*Z*)-3-hexenyl acetate were low at S1 and then increased by about 10- and 8-fold at mature stage S4, respectively. These esters accumulated to even higher levels during ripening at ambient temperature, peaking at the third day of shelf-life (S4 + 3d). Levels of lactones such as γ-decalactone and δ-decalactone were under the detection limit prior to mature S4 stage, accumulating quickly during shelf-life ripening (Figure 1c). These data indicated that green sensory volatiles decrease during peach fruit development and ripening, while fruity and peach-like odor volatiles increase particularly during postharvest stages. Similar patterns of green sensory aldehydes, fruity esters, and peach-like lactones have also been observed by other research groups [21,23]. It has been reported that 18:2 and 18:3 could be used as precursors for production of C6 aldehydes and alcohols using ^14^C-labeling methods [25,26]. The synthesized C6 alcohols are further converted into corresponding esters by alcohol acyltransferase [3]. Therefore, the large changes in volatile profiles prompted us to investigate changes in contents of fatty acids during peach fruit development and ripening [3].

### 2.2. Peach Fruit Fatty Acid Compositions

It has been reported that major fatty acid components in peach fruit are palmitic acid (16:0), stearic acid (18:0), oleic acid (18:1), linoleic acid (18:2), and linolenic acid (18:3) [27]. Our results showed that 18:2 was the major fatty acid in peach fruit (23%–42%), while 18:3 accounted for 5%–20% during development and ripening (Figure 1). Levels of 18:2 were high at S1 and decreased at stone hardening stage S2, followed by a progressive increase during the rest of fruit development and ripening. In contrast, 18:3 declined from 20% at S1 to 8% at S4 and further decreased to 5% at S4 + 3d. Total fatty acid content exhibited a similar profile as that of 18:2 during peach fruit development and ripening (Figure 1d,e). Previous study showed that 18:2 and 18:3 increased over 6 days at 20 °C accompanying post-climacteric ethylene production and loss of firmness [20]. Similar trends in 18:2 and 18:3 were observed in peach fruit by other research groups [28].

Opposite trends of 18:2 and 18:3 indicated that there was a conversion between these two PUFAs during peach fruit development and ripening. Moreover, it has been reported that treatment with 18:2 and 18:3 affected fruit volatile profiles [5,6,7]. Our present work showed that content of 18:2 exhibited a similar pattern with that of hexanal, while content of 18:3 was similar with that of (*E*)-2-hexenal and (*Z*)-3-hexenal as peach fruit developing and ripening (Figure 1). These results prompted us to identify genes that are responsible for synthesis of 18:2 and 18:3 in peach fruit, and then to investigate if these genes are associated with volatile formation.

### 2.3. Cloning and Sequence Analysis of the Peach Fatty Acid Desaturases (FAD) Gene Family

**Figure 2:**
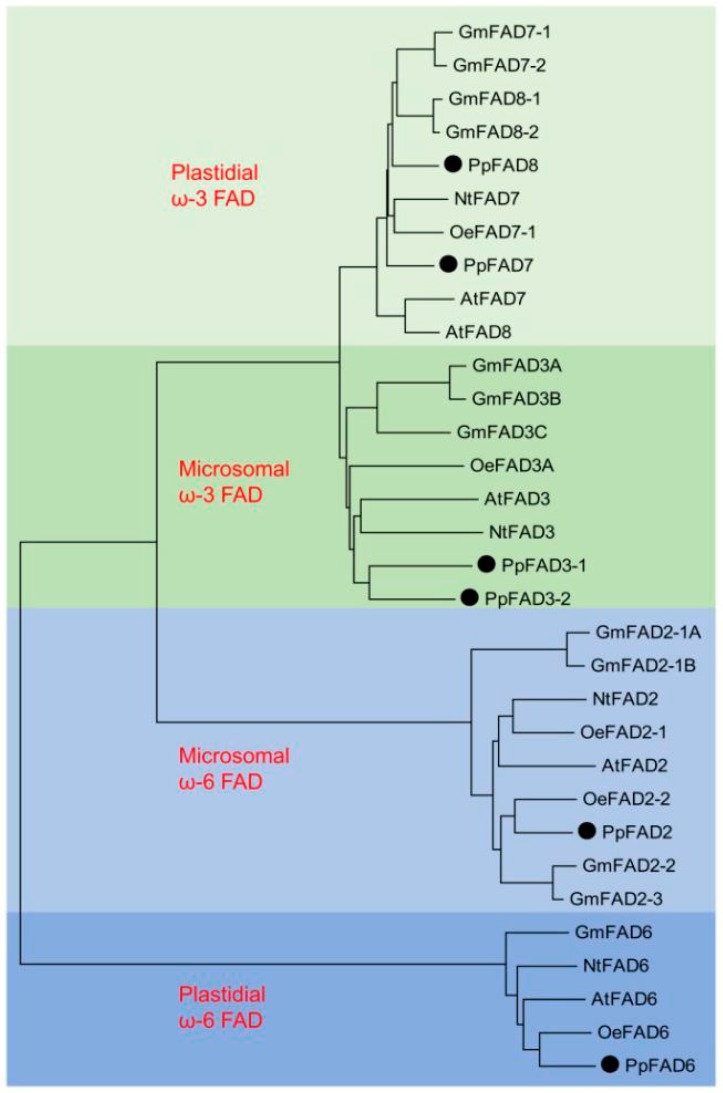
Phylogenetic tree of plant fatty acid desaturases (FAD) showing clustering within the four major FAD groups. Alignments were calculated with ClustalW, and the analysis was performed using the Neighbor-Joining method. FAD groups are indicated by different colors, light green for plastidial ω-3 group, green for microsomal ω-3 group, light blue for microsomal ω-6 group, and blue for plastidial ω-6 group. Peach FADs are shown with black dots.

Synthesis of polyunsaturated fatty acids 18:2 and 18:3 is catalyzed by FAD [9]. Therefore, six candidate FAD genes were cloned based on sequences predicted by the peach genome database, and were named *PpFAD2*, *PpFAD3-1*, *PpFAD3-2*, *PpFAD6*, *PpFAD7*, and *PpFAD8* according to their phylogenetic relationships (Figure 2).

*PpFAD2* clustered with microsomal ω-6 FAD, and *PpFAD6* clustered with plastidial ω-6 FADs. *PpFAD3-1* and *PpFAD3-2* were clustered with microsomal ω-3 FADs. *PpFAD7* and *PpFAD8* were positioned in the group corresponding to plastidial ω-3 FAD enzymes. The deduced amino acid sequence of *PpFAD7* exhibited high similarity to tobacco *NtFAD7* (78%) and olive *OeFAD7-1* (78%). For *PpFAD8*, the deduced amino acid sequence showed 77% identity to soybean *GmFAD8-1*. The deduced amino acid sequences of *PpFAD2* were identical to the previously reported *PpFAD_1B_6* (KC169941) [21]. In addition, *PpFAD2*, *PpFAD3-1*, *PpFAD7*, and *PpFAD6* were identical to *PpFAD1*, *PpFAD2*, *PpFAD3*, and *PpFAD4* in our previous research [20], respectively. For ω-6 FAD, single genes encoding FAD2 and FAD6 were observed in peach, consistent with *Arabidopsis* and tobacco. In the case of microsomal ω-3 FAD, there were two peach isoforms of FAD3. Similar observations have been reported in other plants including olives [29], soybeans [13], and flax [30]. For six peach FADs, the highest 76.94% identity of the nucleic acid sequence was observed between *PpFAD3-1* and *PpFAD3-2*, the lowest 48.42% between *PpFAD2* and *PpFAD8* (Table S1).

### 2.4. Expression of Peach FAD Genes

**Figure 3:**
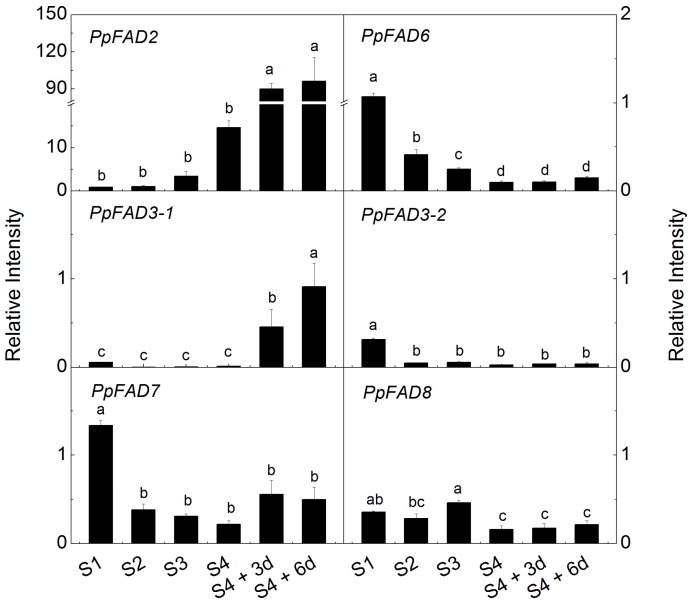
Expression of *PpFADs* during peach fruit development and ripening. Data are the mean ± stand error from three replicates. Values indicated by different letters represent significant difference at level of 0.05.

To study the possible roles of peach FADs associated with formation of PUFAs and derived volatiles, qPCR was performed to determine expression patterns during fruit development and ripening. Transcript levels of *PpFAD2* increased rapidly from S1 to mature stage S4, and further increased ~100-fold during postharvest ripening (Figure 3). The present study showed that transcripts of *PpFAD2* increased as fruit develop and ripen, similar to that of *PpFAD_1B_6* [21] and *PpFAD1* [20], although different cultivars were used for analysis. Fruit ripening induced expression of *FAD2* was also observed in strawberries [31,32]. It has been assumed that *FAD2* expression is associated with fruit esters and lactones production during peach and strawberry fruit ripening [21,31]. An opposite expression pattern was observed for *PpFAD6*, the other peach ω-6 FAD, which had the highest transcript levels at S1 and decreased rapidly at S2 to a constant low level (Figure 3). Decreased transcript accumulation was also observed for *OeFAD6* during olive fruit ripening [16].

Among the four ω-3 FAD genes, *PpFAD3-1* transcript abundance increased noticeably during peach fruit development and ripening. Compared to S4 mature stage, large increases in transcript levels of *PpFAD3-1* were observed after six days of ripening following peach fruit harvest. Increased accumulation of *PpFAD3-1* was in agreement with our previous study during postharvest ripening at ambient temperature [20]. *PpFAD3-2* and *PpFAD7* showed similar decreases during peach fruit development; transcript levels decreased to 15% and 29% at S2 in relation to S1, respectively. Recently, Hernandez *et al.* [29] suggested that *OeFAD3* and *OeFAD7* were responsible for 18:3 present in olive fruit. In the case of *PpFAD8*, the highest transcripts were detected at S3. These results show that the peach FAD genes are temporally regulated during fruit development and ripening. Moreover, active expression of FADs implies strong correlations with changes in contents of 18:2 and 18:3, and concentrations of fruit volatiles such as green sensory aldehydes (Figure 1).

### 2.5. Heterologous Expression of FADs in Tobacco

**Figure 4:**
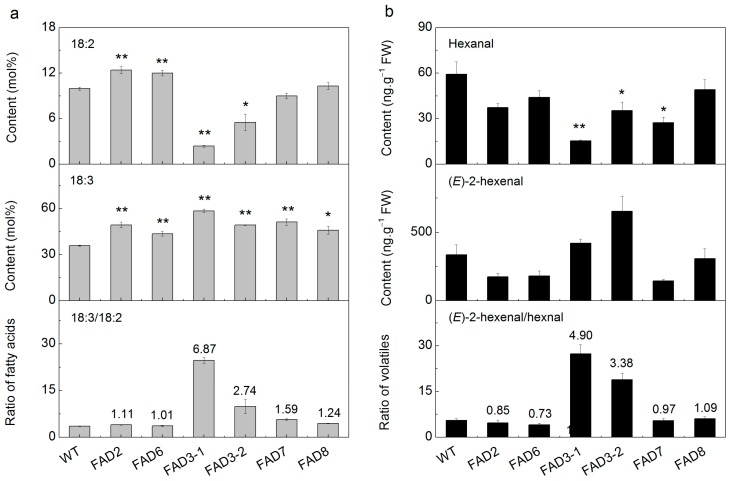
Fatty acid and volatile compositions of transgenic tobacco and wild-type (WT) plant leaves. (**a**) Fatty acid composition; (**b**) volatile production. Relative quantification of volatile compounds was performed using the peak area of the internal standard as a reference. Data are the mean ± stand error from three independent experiments. Significant differences are indicated with asterisks above the bars (* *p* ≤ 0.05 and ** *p* ≤ 0.01). Numbers above the bars represent fold changes relative to WT.

To test if modification of peach fruit FADs expression could alter profiles of FAs and volatiles, particularly the C6 aldehydes, peach FAD genes were transformed into tobacco plants driven by the cauliflower mosaic virus (CaMV) 35S promoter. Three transgenic lines for each gene were obtained and used for further biochemical analysis. Firstly, we tested whether heterologous expression of peach FADs would modify fatty acid composition using tobacco seedlings cultivated on Murashige and Skoog (MS) medium with kanamycin. Transgenic lines overexpressing *PpFAD2* contained significantly higher contents of 18:2 relative to wild-type (WT) plants (Figure 4a). Accumulation of product 18:2 in transformants was consistent with results from heterologous expression in yeast [21], indicating that *PpFAD2* is responsible for 18:2 biosynthesis. For plants transformed with *PpFAD6*, the contents of 18:2 increased by about 21% relative to WT. For ω-3 fatty acid desaturase isoforms, large changes were mainly observed in contents of 18:2 and 18:3. For *PpFAD3-1* transformants, contents of 18:2 were reduced while 18:3 was significantly higher, decreasing by 80% and increasing by 50% in relation to WT, respectively. As a consequence, the ratio of 18:3/18:2 increased considerably in transgenic tobacco (Figure 4a). Similar trends were also detected for *PpFAD3-2*. These results are in agreement with those observed in transgenic tomato where over-expressing FAD3 led to a reduction in 18:2 and accumulation of 18:3 [19]. For tobacco plants transformed with plastidial *PpFAD7*, a similar but less pronounced trend was observed for contents of fatty acids and ratio of 18:3/18:2 (Figure 4a). The transgenic work implied that peach FADs are associated with modification of fatty acid ratio during fruit development and ripening.

Transgenic plants overexpressing FAD genes exhibited modifications in fatty acids such as 18:2 and 18:3. Therefore, we tested if concentrations of hexanal and (*E*)-2-hexenal were modified in transgenic plants. Compared to WT plants, concentrations of hexanal were decreased by about 75%, while (*E*)-2-hexenal increased by about 24% in plants overexpressing *PpFAD3-1* (Figure 4b). These changes in volatile contents were likely caused by the decrease of 18:2 and increase of 18:3. For the other isoforms of peach FAD3, volatile analysis showed a significant reduction in hexanal and a slight increase in (*E*)-2-hexenal in *PpFAD3-2* transformants. Our results are consistent with previous work reporting that overexpression of FAD3 resulted in a significant reduction in hexanal of tomato fruit and leaves [19]. Consequently, the ratio of (*E*)-2-hexenal/hexanal was about 4.90- or 3.38-fold higher than that of WT in *PpFAD3-1* and *PpFAD3-2* transformants, respectively. As expected, these modifications of volatiles were accompanied by considerable changes in the 18:3/18:2 ratio in transgenic plants (Figure 4). Phylogenetic relationships indicated that peach *PpFAD7* and *PpFAD8* encode plastidial ω-3 fatty acid desaturase and heterologous expression of these two genes resulted in marked decrease in hexanal with respect to WT. However, no significant changes in (*E*)-2-hexenal and the ratio of (*E*)-2-hexenal/hexanal were observed in transgenic tobacco plants (Figure 4). In the case of ω-6 fatty acid desaturases, plants overexpressing *PpFAD2* and *PpFAD6* exhibited decreased levels of hexanal but no significant changes in the ratio of (*E*)-2-hexenal to hexanal. Together with the previous study, our results indicate that the reduction of fatty acids rather than oversupply may have a greater effect on production of the derived volatiles [19].

Overexpressing *PpFAD2* and *PpFAD6* did not affect the balance between the (*E*)-2-hexenal and hexanal. However, it is worth noting that over-expressing peach FAD3 caused a larger modification in the 18:3/18:2 ratio and the (*E*)-2-hexenal/hexanal ratio than observed in FAD7 transformants (Figure 4), suggesting different contributions between the microsomal and the plastidial FADs. Such stronger effects caused by FAD3 than FAD7 or FAD8 were also observed in transgenic tobacco [10] and tomato [19]. It has been widely reported that lipids can be transported between the ER and the chloroplast; however, the molecular mechanism of this transfer remains unclear [33]. Nevertheless, results from peach fruit and transgenic tobacco plants suggest that formation of green note C6 aldehydes and are associated with the expression of *PpFAD3*.

## 3. Materials and Methods

### 3.1. Plant Material and Sampling

Melting flesh peach (*Prunus persica* L. Batsch cv. Hujingmilu) fruits were harvested from Ningbo, Zhejiang Province, China. The fruits were harvest at four stages (S1, S2, S3, and S4), representing the first fast growth, endocarp lignification (stone hardening), the second fast growth, and mature stage (ready for harvest), respectively. After harvested at the S4 stage, fruit were allowed to ripen up to 6 days (20 °C, 90%–96% relative humidity), and were sampled at 3 d (S4 + 3d) and 6 d (S4 + 6d). At each sampling time, slices of peel tissue (~1 mm thick) were carefully separately and frozen in liquid nitrogen, then kept at −80 °C until analyzed. Three replicates of five fruit each were used for biochemical and molecular analyses.

### 3.2. Fatty Acid Analysis

Fatty acid composition of peach was analyzed according to Dominguez *et al.* [19]. Total lipids were extracted from 1 g of peach exocarp. Total fatty acids of total lipids were transformed into their corresponding fatty acid methyl ester (FAME) by the addition of 3 mL of methanol:toluene:H_2_SO_4_ (88:10:2, *v*/*v*/*v*). One milliliter of heptane was then added, and the upper phase containing the FAME was analyzed by the gas chromatograph Agilent 6890N (Agilent, Santa Clara, CA, USA) with a flame ionization detector and equipped with a DB-Wax column (0.25 mm, 30 m, 0.25 µm, J & W Scientific, Folsom, CA, USA). Chromatograph conditions were as follows: injection, 230 °C; initial oven temperature 50 increased to 200 °C at 25 °C·min^−1^, increased to 230 °C at 3 °C·min^−1^, and held for 3 min. Nitrogen was used as the carrier gas with 1 mL·min^−1^. Relative quantification of the lipid content of the samples was carried out using the peak area of internal standard heptadecanoic acid (17:0) as a reference. For transgenic tobacco plants, fatty acids were analyzed as described above, but starting materials were 0.2 g of leaves.

### 3.3. GC-MS Analysis of Volatile Compounds

Volatile determination was performed according to Zhang *et al.* [20]. Headspace volatiles were collected from 1 g of peach exocarp and tobacco leaves by using auto solid-phase micro-extraction (SPME) analysis with a fiber coated with 65 µm of polydimethylsiloxane and divinylbenzene (Supelco Co., Bellefonte, PA, USA). Before the vials were sealed, a known amount of 2-octanol was added as an internal standard. The released volatile compounds were separated by a DB-WAX column (0.25 mm, 30 m, 0.25 µm, J & W Scientific). Temperature program was as follows: initial oven temperature started at 40 °C, increased by 3 °C·min^−1^ to 100 °C, and then rose to 245 °C by 5 °C·min^−1^. Helium was used as a carrier gas at a flow rate of 1.0 mL·min^−1^. The column effluent was ionized by electron ionization (EI) at energy of 70 eV with the transfer temperature of 250 °C and the source temperature of 230 °C. Mass scanning was done from 35 to 350 *m*/*z* with a scan time of 7 scans per second. Volatile compounds were identified by comparing with their electron ionization (EI) mass spectra to NIST/EPA/NIH Mass Spectral Library (NIST-08 and Flavor) and retention time of authentic standards (Sigma-Aldrich, St. Louis, MO, USA). Relative quantification of compounds was performed using the peak area of the internal standard as a reference based on total ion chromatogram (TIC).

### 3.4. Sequence Analysis of Peach FAD Genes

FAD sequences of *Arabidopsis* were used as queries to search the peach genome database v2.1 in Phytozome 10.3 (http://phytozome.jgi.doe.gov/pz/portal.html) using BlastP programs with default parameters. Multiple sequence alignments of full-length protein sequences were performed using software MEGA (version 6.0) with default parameters [34]. The Neighbor-Joining phylogenetic tree was constructed using Poisson correlation model with 1000 bootstrap replications. Accession number of the different FADs are: *Arabidopsis thaliana* (*AtFAD2*, NM_112047; *AtFAD3*, NM_128552; *AtFAD6*, NM_119243; *AtFAD7*, NM_111953; *AtFAD8*, NM_120640); *Nicotiana tabacum* (*NtFAD2*, AY660024; *NtFAD3*, D26509; *NtFAD6*, KJ551513; *NtFAD7*, D79979); *Glycine max* (*GmFAD2-1A*, L43920; *GmFAD2-1B*, AB188251; *GmFAD2-2*, L43921; *GmFAD2-3*, DQ532371; *GmFAD6*, L29215; *GmFAD3A*, AY204710; *GmFAD3B*, AY204711; *GmFAD3C*, AY204712; *GmFAD7-1*, GQ144962; *GmFAD7-2*, EU621390; *GmFAD8-1*, FJ393229; *GmFAD8-2*, NM_001252848); *Olea europaea* (*OeFAD2-1*, AY733076; *OeFAD2-2*, AY733077; *OeFAD3A*, DQ788673; *OeFAD6*,AY733075; *OeFAD7-1*, DQ788674); *Prunus persica* (*PpFAD2*, XM_007204334; *PpFAD6*, XM_007203193; *PpFAD3-1*, XM_007205323; *PpFAD3-2*, XM_007205280; *PpFAD7*, XM_007217933; *PpFAD8*, XM_007205113).

### 3.5. RNA Extraction and qPCR Analysis

Total RNA was isolated from approximately 2 g of frozen peach fruit exocarp according to the protocol described by Zhang *et al.* [20] followed by treatments with TURBO DNA-*free*^TM^ kit (Ambion, Hopkinton, MA, USA) to remove the possible contaminating genomic DNA. The first strand cDNA was synthesized using iScript™ cDNA synthesis kit (Bio-Rad, Hercules, CA, USA). The *PpTEF2* was used as the internal control to normalize small differences in template amounts according to Tang *et al.* [35]. For tobacco, total RNA was extracted from leaves using TRIzol™ (Invitrogen, Waltham, MA, USA), and the internal control was *NtEF1-α* [36]. Oligonucleotide primers for qPCR were designed by Primer 3.0 and listed in Table 1. The qPCR mixture (20 µL total volume) comprised 10 µL SsoFast Eva Green supermix (Bio-Rad), 1 µL of each primer (10 µM), 2 µL of diluted cDNA, and 6 µL of PCR-grade H_2_O, and the reaction was performed on a Bio-Rad CFX96 instrument. The temperature program was as follows: initiated at 95 °C for 3 min, followed by 45 cycles of 95 °C for 10 s, 60 °C for 30 s, then completed with a melting curve analysis program.

### 3.6. Tobacco Transformation, Growth Conditions and Sampling

Full length open reading frame of six peach FAD genes were amplified with particular primers (Table S2) and then were cloned to pGreen 0029 62 SK (EU048865), a binary vector with CMV35S promoter. The recombined vectors were verified via sequencing and then were transformed to *Agrobacterium tumefaciens* strain GV3101::pSoup. Tobacco transformation was performed as described by Huang *et al.* [37]. After identifying the ability to grow on kanamycin-containing medium, potential rooted transformants were transferred to artificial climate chambers (25 °C, 16 h light/8 h darkness). Putative transgenic plants were screened via PCR (Table S3) and qPCR (Table S4) analysis. Seedlings of T1 generation of three lines for each peach FAD gene grew on solid MS medium with 150 mg·L^−1^ kanamycin for one month. Three lines with 8 to 10 seedlings each were sampled for further molecular and biochemical analysis.

### 3.7. Experimental Design and Statistical Analysis

A completely randomized design was used in the experiments. OriginPro 8.6 (OriginLab Corporation., Northampton, MA, USA) was used to prepare figures. Duncan’s test was used for ANOVA to detect significant differences among groups at significant level of 0.05. For the two-sample comparison, an unpaired Student’s *t*-test was applied to examine the significance. Statistical analysis was performed via SPSS 19.0 software (SPSS Inc., Chicago, IL, USA).

## 4. Conclusions

Six FAD genes were cloned from peach, and heterologous expression in tobacco plants resulted in modifications in fatty acid composition, indicating that these genes are involved in desaturation of fatty acids. Expression of *PpFAD2* and *PpFAD3-1* increased during development and ripening while that of other genes decreased, suggesting temporal regulation of peach FAD genes. Transgenic work in tobacco, together with correlation between FAD expression levels and volatile concentrations, suggests that *PpFAD3-1* and *PpFAD3-2* are involved in controlling the balance between hexanal and hexenal, therefore affecting the green note of peach fruit.

## Figures and Tables

**Table 1:** Primers used for *PpFADs* expression analysis in peach fruit.

Genes	Forward Primers (5′–3′)	Reverse Primers (5′–3′)
*PpFAD2*	CGGTTTTCAAGGCAATGTTC	CCTACACTCATTCGGGCAAT
*PpFAD6*	ACGTTGCCTTTGACCAACTT	AATGACTGTGACCCCACCAC
*PpFAD3-1*	AGTGACACAGGAGATATTGTGT	TTCGAAAGATTACGAGGATTTCA
*PpFAD3-2*	ATTGAGATGGCAGGGATGAA	CCTTCTCAAGGTTTTTCAGCA
*PpFAD7*	TCAGGCACAACAATTGAAGC	AAGAATGGCTGCCCATACAG
*PpFAD8*	TGACCACAAAGACAACCTTTCA	ACTAGGGCACCACCCTTTTT

## Supplementary Materials

Supplementary materials can be found at http://www.mdpi.com/1422-0067/17/4/464/s1.
