# Supplementary material for: Two ω-3 FADs Are Associated with Peach Fruit Volatile Formation
Source: Int J Mol Sci. 2016 Mar 29;17(4):464. doi: 10.3390/ijms17040464 (PMC4848920; doi:10.3390/ijms17040464)
Supplement: Supplementary file 1 [file ijms-17-00464-s001.pdf]

Supplementary Materials: Two ω-3 FADs Are Associated with Peach Fruit Volatile Formation

Jiao-Jiao Wang, Hong-Ru Liu, Jie Gao, Yu-Ji Huang, Bo Zhang and Kun-Song Chen

Table S1. Nucleic acid sequence identity between peach six fatty acid desaturases (FADs).

Genes	*PpFAD2*	*PpFAD3-1*	*PpFAD3-2*	*PpFAD6*	*PpFAD7*	*PpFAD8*
PpFAD2	1	–	–	–	–	–
PpFAD3-1	52.08%	1	–	–	–	–
PpFAD3-2	50.63%	76.94%	1	–	–	–
PpFAD6	49.02%	51.16%	53.58%	1	–	–
PpFAD7	52.60%	70.32%	73.43%	52.11%	1	–
PpFAD8	48.42%	70.73%	72.32%	48.77%	76.30%	1

Table S2. Primers used for amplifying the ORFs of *PpFAD*s.

Genes	Primers′ Name	Sequence (5′–3′)
PpFAD2	PpFAD2-SK-FP	TATGGATCCATGGGTGCCGGTGGAAGAAT
	PpFAD2-SK-RP	CGCGAATTCCGGGCAATTTTGATAATCAC
PpFAD6	PpFAD6-SK-FP	CGTGGATCCATTTCTGCAATTTCTGCTC
	PpFAD6-SK-RP	GGCGAATTCTCAAGCATAATCAGGCATC
PpFAD3-1	PpFAD3-1-SK-FP	TCAGGATCCATGGAGACTAGTGTGACCAG
	PpFAD3-1-SK-RP	GCCGAATTCTTACGAGGATTTCATCTTCTC
PpFAD3-2	PpFAD3-2-SK-FP	ATATCTAGAATGGTGGAGGCTCAGAGCA
	PpFAD3-2-SK-RP	GGCGAATTCTCAACTAGACTTGCTCTTAAG
PpFAD7	PpFAD7-SK-FP	ATAGGATCCATGGCAAGTTGGGTTCTCTC
	PpFAD7-SK-RP	GGCGAATTCTCATTCTGATGTCACAGAC
PpFAD8	PpFAD8-SK-FP	TTATCTAGAATGGCGGCGAGTTGGGTTC
	PpFAD8-SK-RP	GGCGAATTCGGTTGTCTTTGTGGTCATC

Table S3. Primers used for identifying transgenic tobacco plants overexpressing *PpFADs*.

Genes	Primers′ Name	Sequence (5′–3′)
pGreen 0062 SK	SK-FP	AATCCCACTATCCTTCGCAAGACCCTTC
	SK-RP	AGAGACTGGTGATTTCAGCGAATTGGTAC

Table S4. Primers used for detecting expression of *PpFADs* in transgenic tobacco plants.

Genes	Primers' Name	Sequence (5′–3′)
PpFAD2	TG-PpFAD2-FP	AGAACGCTGAAACCGACAAC
	TG-PpFAD2-RP	GTCACCCTCATCTCGCTCAAC
PpFAD6	TG-PpFAD6-FP	CATTGGTTGCTAGTCCACTTCG
	TG-PpFAD6-RP	GGCCATCCAATCCCCATAAAC
PpFAD3-1	TG-PpFAD3-1_FP	TTATGGCTGCTTTGCTTTTCC
	TG-PpFAD3-1_RP	GTATGTGACAATGTCCAACCAC
PpFAD3-2	TG-PpFAD3-2_FP	ATGAGTCTTGGGTTCCTCTGAC
	TG-PpFAD3-2_RP	GAGAACCTTGCTTTCCTGGAC
PpFAD7	TG-PpFAD7-FP	GAACGGCCTAACATCAAAGAC
	TG-PpFAD7-RP	GCCCAATTTGTCCCTCGAATC
PpFAD8	TG-PpFAD8-FP	ACCATGACATAGGCACCCATG
	TG-PpFAD8-RP	CAAGCAGGTGAAACGGTAGAG
NtEF1α	NtEF1α-FP	GCCCAACACTTCTTGATGCTC
	NtEF1α-RP	GACACCAGTTTCCACACGAC
